# Efficacy of sinus crestal elevation with demineralized dentin matrix at the apex of the implants

**DOI:** 10.1186/s40729-026-00681-7

**Published:** 2026-04-21

**Authors:** Jung-Hyun Kwon, Yesel Kim, In-Woong Um, Pil-Young Yun, Jeong-Kui Ku

**Affiliations:** 1 Department of Oral and Maxillofacial Surgery, Section of Dentistry, Seoul National University Bundang Hospital, 300 Gumi-dong, Bundang-gu, Seongnam, Gyeonggi-do 13620 Republic of Korea; 2 Electronic Claims Review Division, Claims Review Standards Department, Health Insurance Review & Assessment Service, Wonju, 26465 Republic of Korea; 3 R&D Institute, Korea Tooth Bank, Seoul, 06101 Republic of Korea; 4 Department of Dentistry and Dental Research Institute, School of Dentistry, Seoul National University, 03080 Seoul, Republic of Korea

**Keywords:** Bone regeneration, Dental implants, Demineralized dentin matrix, Sinus floor augmentation, Maxillary sinus

## Abstract

**Background:**

This study evaluated the clinical and radiographic outcomes of using demineralized dentin matrix (DDM) as a graft material at the implant apex during crestal sinus floor elevation. We hypothesized that DDM would promote predictable bone regeneration at the sinus floor and facilitate implant stability with minimal surgical morbidity.

**Methods:**

A retrospective analysis was performed on 55 implants placed with transcrestal sinus floor elevation and DDM grafting at the implant apex in the posterior maxilla. Radiographic outcomes were assessed with CBCT. The primary variable was vertical bone gain at the former sinus floor. Secondary outcomes included the proportion of implant protrusion length replaced by new bone (bone gain), implant survival, and sinus health. Pearson correlation and t-tests were used to analyze relationships between implant protrusion length, sinus width, and bone gain.

**Results:**

All 55 implants remained functional and osseointegrated after a mean follow-up of 48.1 ± 32.7 months, yielding a 100% survival rate. The mean implant protrusion into the sinus was 3.42 ± 1.27 mm, and mean bone gain at the sinus floor was 1.97 ± 1.18 mm. On average, 61.5% of the protruded implant length was covered by new bone. Implants protruding ≥ 4 mm showed significantly greater absolute bone gain than those < 4 mm (2.50 vs. 1.65 mm, *p* = 0.04), although the bone fill percentage was similar between groups. Implants in narrower sinuses exhibited higher bone fill (77.8% vs. 46.8%, *p* < 0.01) and greater bone gain than those in wider sinuses. No significant sinus complications or graft failures occurred.

**Conclusion:**

Within the limitations of this retrospective study, crestal sinus elevation with DDM grafting at the implant apex proved to be a safe and effective minimally invasive technique, consistently yielding bone regeneration and excellent implant survival in the posterior maxilla.

## Background

Sinus floor elevation has become a routine procedure in implant dentistry to enable implant placement in the atrophic posterior maxilla. Two principal approaches are used: the lateral (window) approach and the crestal (transcrestal) approach [1]. Various techniques and instruments have been developed to achieve effective and safe sinus surgery [2]. The lateral window technique allows larger augmentation volumes even in extremely pneumatized sinuses, but it is more invasive [3, 4]. Since the 1990s, several modifications of the crestal approach have been introduced (e.g., osteotome sinus floor elevation [OSFE] and bone-added OSFE). However, these early osteotome-based techniques had drawbacks that the need for malleting could cause patient discomfort or vertigo, and Schneiderian membrane perforation was relatively common due to limited tactile control. Membrane perforation is the most frequent complication of sinus augmentation, occurring in roughly 10–60% of cases, which can lead to graft migration or sinusitis [5–7]. Preservation of an intact Schneiderian membrane is therefore critical for uneventful healing and graft success [8]. 

In recent years, newer devices and methods have been developed that allow a safer crestal approach. In general, successful outcomes can be achieved with sinus floor elevation as long as key conditions are met including absence of sinus pathology, a healthy Schneiderian membrane, sufficient residual bone height, and no membrane perforation during surgery [9–11]. Atraumatic crestal elevation techniques (e.g., using hydraulic pressure or specialized drills) aim to lift the sinus lining gently to reduce perforation risk. Notably, some studies have shown that substantial bone formation can occur beneath an elevated but ungrafted sinus membrane, relying only on the blood clot and the membrane’s osteogenic potential [12, 13]. The Schneiderian membrane has demonstrated innate osteogenic capability, contributing to new bone formation in the elevated sinus floor compartment – although this effect is weaker than that of native bone walls [14]. Clinically, this translates to the possibility of achieving implant integration without graft material if the membrane remains intact. For example, a recent case series using a crestal approach with no graft reported about 2.4 mm of new bone formation after one year when implants protruded at 4.0 mm into the sinus, alongside a 100% implant survival rate [12]. Similarly, other researchers have observed ~ 73% spontaneous bone fill in cystic cavity defects after 1–2 years, suggesting the tenting effect of an intact membrane can facilitate bone regeneration [15]. 

Despite these encouraging findings, leaving the implant apex entirely unencased by bone (i.e. in direct contact with the sinus cavity and membrane) may raise concerns about long-term outcomes. Implants protruding into an ungrafted sinus might develop fibrous tissue encapsulation at the apex or be at risk for late compromise if sinus pathology occurs [16–18]. Therefore, many clinicians favor placing a small amount of graft material at the implant apex even in crestal approaches, to serve as an osteoconductive scaffold and perhaps expedite bone coverage of the implant. Demineralized Dentin Matrix (DDM) has emerged as a promising autogenous graft material in this context. DDM is derived from the patient’s own extracted teeth, processed to remove the mineral phase while exposing collagen and growth factors such as BMPs [19]. It has been shown to have excellent osteoinductive properties and to behave similarly to autogenous bone graft without the need for a secondary donor site [20]. We hypothesized that placing demineralized dentin matrix at the apex of implants during a crestal sinus elevation would promote reliable bone formation around the implant tip without the need for a lateral window approach. The present study aimed to evaluate the radiographic bone gain and clinical outcomes of implants protruding into the sinus when a DDM graft is applied to the implant apex via a crestal approach.

## Methods

This study was conducted in accordance with the ethical guidelines of the Declaration of Helsinki and was approved by the Institutional Review Board of Seoul National University Bundang Hospital (IRB No. B-2510-1001-104).

This was a retrospective observational study of patients between February 2015 and January 2025 who underwent transcrestal sinus floor elevation with simultaneous implant placement in the posterior maxilla, using demineralized dentin matrix as graft material at the apex of the implant. All cases had insufficient residual vertical bone in the maxillary posterior region and thus required sinus augmentation for implant placement. Inclusion criteria were: posterior maxillary edentulism with necessary to sinus bone graft, healthy sinus (no active sinusitis or cystic lesion), an intact Schneiderian membrane at surgery, and follow-up radiography at least 3 months after the loading. Exclusion criteria were any sinus pathology or history of sinus surgery, use of any bone graft materials or membranes other than DDM, cases where the DDM graft was not radiographically visible at the implant apex after healing, and uncontrolled systemic conditions that could impair healing.

The extracted hopeless teeth were processed at a certified facility (Korea Tooth Bank, Seoul, Republic of Korea) in accordance with the Good Practice Guidelines for Tooth Handling Institutions established by the Korean Ministry of Health and Welfare [21]. Preparation of demineralized dentin matrix (DDM) involved initial preservation in 70% ethanol, followed by meticulous removal of soft tissue and pulp through a retrograde cleansing procedure. The cleaned dentin was subsequently crushed into granules sized 300–800 μm, degreased, and demineralized using 0.6 N hydrochloric acid. A viral inactivation step was additionally performed following the protocol described in Patent EP 2,601,982 [22]. 

**Fig. 1:**
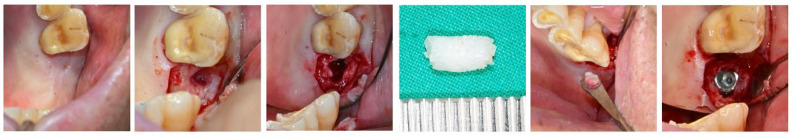
Intraoperative view of the crestal sinus floor elevation procedure. After flap reflection, the maxillary sinus floor is accessed via the edentulous ridge. The osteotomy for the sinus lift is prepared at the second premolar site, and a demineralized dentin matrix (DDM) graft is placed at the osteotomy apex before implant insertion

All surgeries were performed by expert oral and maxillofacial surgeons. After a mid-crestal incision and flap reflection, the implant osteotomy was prepared up to approximately 1 mm short of the sinus floor using sequential drills. A specialized sinus osteotome elevation system (traditional sinus crestal elevation, Summer’s osteotome, AERO drill) was then used to gently lift the sinus floor and detach the sinus membrane without creating a lateral window. Once the Schneiderian membrane was raised, DDM particulate was placed on the drilling site. (Fig. 1) Immediately thereafter, a dental implant (Tapered and SLA surfaced internal connection type; TSIII, Osstem Implant; SuperLine, Dentium; IS-III Active, Neobiotech; One Day Implant, Oneday Biotech) was inserted into the site. The implant itself helped push the membrane further upward (providing a tenting effect) and also served to contain the graft material at the apex area. If good primary stability was achieved (insertion torque ≥ 30 Ncm or ISQ (Osstell ≥ 60), a one-stage approach was used. If primary stability was lower, a two-stage protocol was used with the implant submerged and allowed to heal for about 5 months. The flaps were sutured for primary closure, and patients were prescribed routine postoperative antibiotics, decongestants, and instructed to follow standard sinus precautions.

The implants were generally restored the prosthetics after a conventional healing period (approximately 3–5 months). Follow-up CBCT scans were obtained at least 3 months after functional loading (ranging from 8 to 120 months postoperatively), to assess bone regeneration at the sinus floor. The primary outcome measure was the vertical bone gain at the former sinus floor, defined as the height of new bone formed between the original sinus floor and the most apical point of the implant in contact with new bone.

**Fig. 2:**
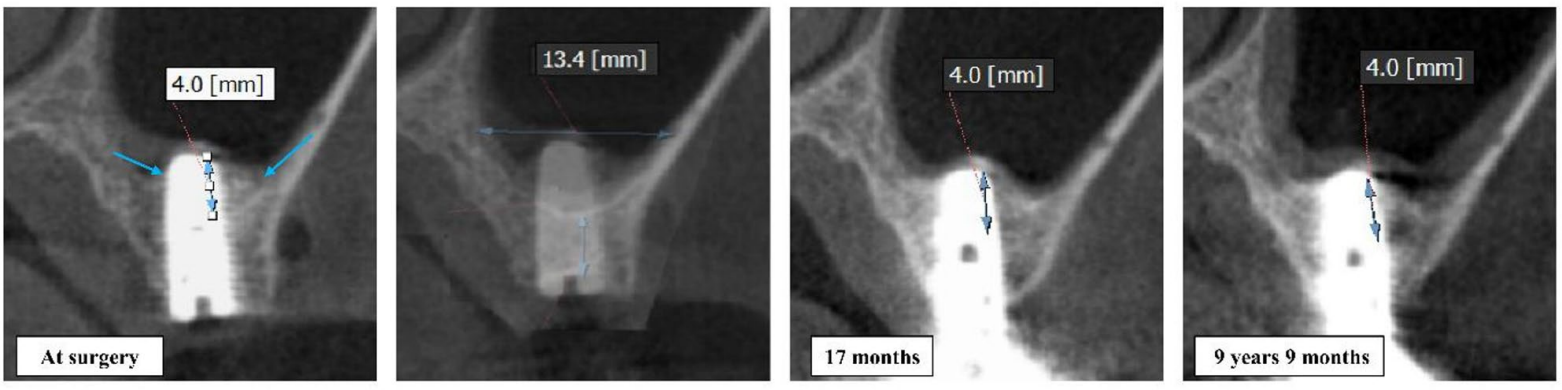
Method for measuring vertical bone gain. The vertical distance from the original sinus floor to the new bone–implant and the sinus width at the level of the implant apex was measured on CBCT cross-sections

This distance was measured on cross-sectional CBCT images as the distance from the original bony sinus floor to the new bone–implant interface at the implant apex. (Fig. 2) The original sinus floor was identified from preoperative imaging or surgical records. The primary outcome was the vertical bone gain at the sinus floor, defined as the height of new bone formed between the original sinus floor and the most apical point of the implant that was in contact with new bone on follow-up imaging. This distance was measured on cross-sectional CBCT views (or calibrated panoramic images) as the distance from the original bony sinus floor up to the new bone-implant interface at the apex. The original sinus floor reference was determined from preoperative imaging or surgical records. The extent of implant protrusion into the sinus (the length of implant extending beyond the original bone level) was recorded for each implant at placement. The sinus width was defined as the horizontal distance between the buccal and palatal walls measured at the level of the implant apex on cross-sectional CBCT images. (Fig. 2) Secondary outcomes included the percentage of the implant’s protruding length that became filled with new bone (i.e., bone gain height divided by protrusion length, expressed as a percentage), changes in sinus health (any signs of sinusitis or mucosal thickening on follow-up radiographs), and implant success/survival over time. Any intraoperative or postoperative complications (e.g., sinus membrane perforation, persistent sinus infection, graft extrusion, implant failure) were also documented.

All radiographic measurements were performed by two calibrated examiners, and the values were averaged for analysis. For CBCT measurements, the bone heights from the buccal and palatal aspects were averaged to obtain a single value for each implant. Descriptive statistics (mean ± standard deviation) were calculated for all quantitative variables. Pearson’s correlation analysis was used to assess the relationship between the implant protrusion length and the bone gain height. For subgroup comparisons, unpaired two-sample t-tests were conducted (e.g., comparing outcomes between implants protruding < 4 mm vs. ≥4 mm into the sinus, and between “narrow” vs. “wide” sinuses based on the median sinus width). A two-sided significance level of 0.05 was applied for all tests.

## Results

A total of 55 implants (46 Male, 19 Female, 64.6 ± 9.6 years) were analyzed, with a mean follow-up time of 48.1 ± 32.7 months. All 55 implants remained osseointegrated and functional at the last follow-up, yielding a 100% survival rate in this cohort. (Table 1) Patients did not report any sinus-related postoperative complications. Notably, there were no cases of hematoma or radiographic signs suggestive of sinus membrane perforation on postoperative CBCT imaging, and no patients exhibited symptoms of chronic sinusitis or graft rejection. Two patients (3.6%) experienced mild transient sinus congestion during the first postoperative week, which resolved with conservative management. No instances of graft material extrusion into the sinus or sinus cyst formation were observed.

Radiographic analysis confirmed a distinct radiopaque area at the implant apex in all cases, indicating that the implant tip was not in direct contact with the Schneiderian membrane. The mean implant protrusion at placement was 3.42 ± 1.27 mm. After the healing period, the mean vertical bone gain achieved at the former sinus floor was 1.91 ± 1.18 mm. (Table 1) In other words, on average about two-thirds of the implant’s protruding length became covered by new bone. This corresponds to a mean “bone fill” percentage of approximately 61.5% of the sinus-exposed portion of the implant being replaced with bone. Even implants with minimal initial bone support showed some degree of new bone fill at the apex on follow-up radiographs. The average sinus cavity width in this cohort was 16.8 ± 3.2 mm, and the mean thickness of the sinus floor’s buccal and palatal bony walls was 2.74 ± 2.12 mm on each side.

**Table 1:** Patient information and general characteristics

Age (Years)	64.6 ± 9.6
Sex (Male:Female)	46:19
Protrusion (mm)	3.42 ± 1.27
Sinus width (mm)	16.8 ± 3.2
Buccal bone thickness (mm)	2.74 ± 2.12
Palatal bone thickness (mm)	2.74 ± 2.12
Bone gain (mm)	1.91 ± 1.18
Bone healing percent (%)	61.45 ± 43.83
Follow-up (months)	48.1 ± 32.7

When comparing subgroups, implants that protruded further into the sinus tended to induce more new bone formation in absolute height. Implants with ≥ 4 mm of sinus protrusion showed a significantly greater mean bone gain than those with < 4 mm protrusion (2.45 ± 1.49 mm vs. 1.61 ± 0.84 mm, respectively; *p* = 0.029). However, the fraction of the implant length that converted to bone (bone fill percentage) did not differ significantly between these two groups. (Table 2)

Sinus anatomy also appeared to influence the outcome. Using the median sinus width of 16.8 mm to define “narrow” vs. “wide” sinuses, the narrower sinuses exhibited not only a significantly higher bone fill percentage (77.8% vs. 46.8%, *p* = 0.008) but also a greater absolute bone gain height (2.30 ± 1.25 mm vs. 1.57 ± 1.01 mm, *p* = 0.021) compared to the wider sinuses. The narrow sinus subgroup was on average older than the wide sinus subgroup (68.4 ± 7.3 years vs. 61.1 ± 10.2 years, *p* = 0.004). Other measured local variables (such as the residual buccal and palatal bone wall thicknesses) did not show statistically significant differences between these subgroups. (Table 2)

**Table 2:** Comparison of outcomes between subgroups defined by implant protrusion (< 4 mm vs. ≥ 4 mm) and sinus width (< 16.8 mm vs. ≥ 16.8 mm)

	Protrusion			Sinus width		
	< 4 mm (*n* = 35)	≥ 4 mm (*n* = 20)	*p*-value	< 16.8 mm (*n* = 26)	≥ 16.8 mm (*n* = 29)	*p*-value
Age (Years)	65.0 ± 9.8	63.8 ± 95	0.529	68.4 ± 7.3	61.1 ± 10.2	0.004
Sex (Male: Female)	24:11	12:8	0.647	13:13	23:6	0.024
Protrusion (mm)	2.66 ± 0.83	4.74 ± 0.68	< 0.001	3.29 ± 1.21	3.53 ± 1.33	0.498
Sinus width (mm)	17.06 ± 2.91	16.38 ± 3.77	0.748	14.09 ± 1.74	19.26 ± 2.09	< 0.001
Buccal bone thickness (mm)	3.13 ± 2.53	2.12 ± 1.03	0.093	2.40 ± 1.19	3.06 ± 2.72	0.189
Palatal bone thickness (mm)	3.76 ± 1.51	2.63 ± 1.59	0.289	3.66 ± 1.86	3.01 ± 1.33	0.157
Bone gain (mm)	1.61 ± 0.84	2.45 ± 1.49	0.029	2.30 ± 1.25	1.57 ± 1.01	0.021
Bone fill percent (%)	67.29 ± 49.26	51.22 ± 30.76	0.194	77.76 ± 53.60	46.81 ± 25.91	0.008
Follow-up (months)	44.5 ± 26.0	54.5 ± 42.0	0.344	51.4 ± 32.7	45.1 ± 33.0	0.485

**Fig. 3:**
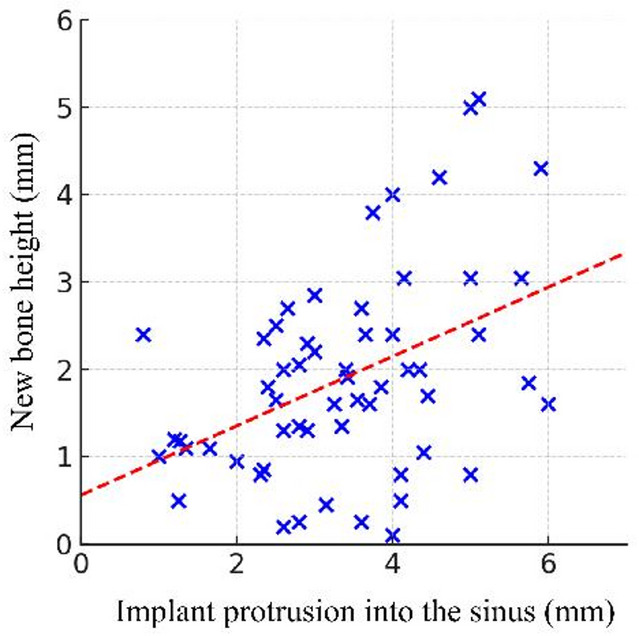
Correlation between implant protrusion length and new bone height at the implant apex. A scatter plot demonstrates a moderate positive correlation (*r* = 0.45, *p* < 0.01) between the length of implant protrusion into the sinus and the amount of vertical bone gain achieved at the former sinus floor. Implants with greater protrusion tended to produce more new bone at the apex, supporting the concept of the tenting effect under the elevated Schneiderian membrane

In addition, there was a moderate positive correlation between the implant protrusion length and the height of new bone formed (Pearson *r* = 0.45, *p* < 0.01). This indicates that, within the range of protrusion lengths in our study, implants extending further into the sinus tended to generate a greater absolute amount of new bone at the apex. Essentially, the available space for bone regeneration (created by implant protrusion) was roughly proportionally filled with new bone in many cases. This relationship supports the concept that a larger tenting space can yield more bone formation (Fig. 3), although complete fill of the space was not achieved in all cases. (typically around 50–60% fill)

## Discussion

This study evaluated the outcome of using DDM graft at the implant apex during a crestal sinus floor elevation. Overall, even a small DDM graft placed via the transcrestal approach effectively promoted bone regeneration around the implant apex. New bone formation was consistently seen on radiographs, confirming the osteogenic potential of the sinus membrane and graft combined. This finding aligns with prior reports of endo-sinus bone gain in graftless approaches [12], and suggests that the presence of the DDM graft at the implant apex did not impede the natural bone healing process under the sinus membrane. In our series, the DDM graft may have also acted as a buffer between the implant tip and the Schneiderian membrane, potentially preventing direct membrane contact and reducing the risk of late membrane perforation or irritation [16]. All implants in our cohort successfully achieved and maintained osseointegration with no adverse sinus events over an average of four years of follow-up.

One notable observation was that the extent of bone gain was roughly proportional to the length of implant protrusion into the sinus. Our data showed that approximately 50–60% of the space created by the protruding implant was healed with newly formed bone across a range of protrusion lengths. (Table 2) This is consistent with the concept of a self-limiting “internal scaffold” under the sinus membrane. The blood clot that forms in the tented space can organize and mineralize to a certain degree, but complete bony fill of the void is not always attained [23, 24]. Additionally, sinus anatomy was found to influence the regenerative outcome. In our study, a smaller sinus cavity width was associated with a greater degree of new bone formation: implants in narrower sinuses (below the median width; 16.4 mm) achieved a higher fraction of vertical bone gain. (Table 2) This phenomenon can be explained by the more confined space in a narrow sinus, where the elevated membrane and graft are in closer contact with surrounding bony walls, providing abundant osteogenic cells and a stable environment for the blood clot to ossify.

**Fig. 4:**
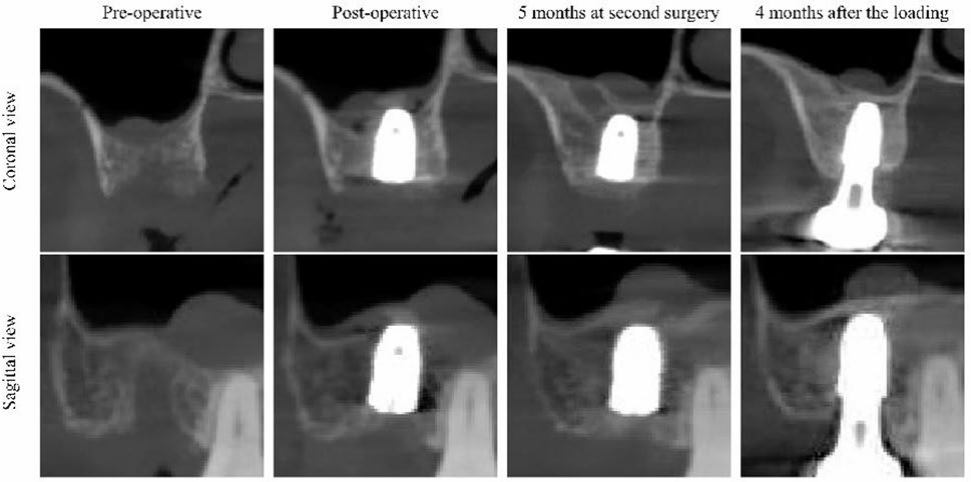
Representative radiographic image showing extensive bone formation at the implant apex. Cross-sectional CBCT view demonstrates that new bone has formed beyond the original grafted DDM area, completely covering and integrating over the graft particles

In other words, simply adding graft material does not guarantee 100% bone fill up to the tip of the implant. the addition of DDM did not dramatically increase the maximum achievable bone height beyond what the membrane-and-blood-clot alone would typically yield; however, DDM did confer other potential benefits. DDM is known to be osteoinductive; it releases growth factors (such as BMPs) as the dentin matrix gradually demineralizes, and its collagenous matrix provides a scaffold favorable for cellular migration and osteogenesis [25]. By placing DDM at the sinus floor, we aimed to accelerate and enhance the bone formation process by both supporting the initial blood clot and providing osteogenic signals. The results suggest that the DDM graft can indeed participate in new bone formation, as evidenced by the consistently formed bone at the apex and a moderate correlation between protrusion depth and bone gain. Histologic and clinical studies have shown that DDM results in new bone that is similar in quality to autogenous bone grafts [25–28]. Furthermore, a recent systematic review concluded that autogenous tooth graft material (such as DDM) is as effective as other graft substitutes in sinus lift procedures, demonstrating excellent biocompatibility and bone regeneration capacity [25]. In our series, the osteoinductive capacity of DDM may have, in some cases, led to greater-than-expected bone growth—where new bone extended beyond the initial tented space provided by the implant and graft. (Fig. 4)

Another consideration is the long-term fate of graft material and the maintenance of the augmented volume. Xenograft materials maintain volume but integrate slowly, often leaving residual particles [29]. Among cases of trephine biopsy the excellent bony remodeling was found only 13.1% out of 153 xenogenic bone grafts [30]. In contrast, DDM is gradually resorbed and replaced by the patient’s own bone, which may promote more complete healing of the sinus floor. This biologic turnover could be advantageous for long-term healing and remodeling of the sinus floor. Indeed, in our patients, follow-up imaging after a few years showed the augmented sinus floor region to be filled with homogeneous bone without any discernible graft particles. Implant survival in our study was 100%, which is consistent with the literature reporting high implant survival rates for both lateral-window and crestal sinus augmentation techniques, regardless of graft type, as long as primary stability of the implants is achieved [20]. From a sinus health perspective, the use of DDM at the implant apex appeared to be very well tolerated. We observed no instances of persistent sinus mucosal inflammation, graft migration, or sinus pathology attributable to the graft. This mirrors the findings of Kim et al., who reported that DDM caused minimal inflammatory response when placed in maxillary sinus augmentation sites, even when in direct contact with the Schneiderian membrane [31]. The excellent biocompatibility and hydrophilicity of DDM, along with its tendency to integrate quickly with the blood clot, likely contributed to the absence of adverse sinus reactions [25, 32]. Additionally, because DDM is an autogenous material, it carries no risk of immune rejection or disease transmission, unlike allografts or xenografts [33, 34]. In addition, the average processing time for DDM preparation was approximately one to two weeks, with a cost comparable to commercially available xenograft materials. These factors make DDM a particularly appealing graft choice for sinus lift procedures. Although our outcomes were uniformly successful with no infections or implant failures, it is important to acknowledge potential complications inherent to the crestal sinus lift technique. Sinus membrane perforation remains a possibility during any crestal approach. If a large tear occurs, graft material (even DDM) could escape into the sinus or cause a reaction. Fortunately, with careful technique, we did not encounter any membrane perforations in this series; however, clinicians should always be prepared to manage a tear, by aborting graft placement or converting to a lateral approach if necessary.

This study has several limitations. First, it was a retrospective analysis with no control group. In the absence of a non-graft control group, we cannot quantitatively prove that the DDM graft resulted in more bone formation or faster healing than would have occurred with a blood clot alone. Second, the follow-up times and imaging modalities in our sample were not uniform; not all patients had CBCT scans at identical time points, which introduces heterogeneity in the assessment of bone outcomes. However, previous evidence suggests that spontaneous sinus bone healing generally stabilizes within approximately 12 months [15]. Therefore, our mean follow-up of 48 months (range, 8–120 months) provides clinically meaningful long-term data. Third, there is also possible selection bias. Because this retrospective cohort primarily included cases with moderate residual bone height suitable for a one-stage approach, selection bias toward less atrophic conditions cannot be excluded. Lastly, although the radiographic evidence of new bone formation is strong, we did not obtain histologic confirmation of the nature of the newly formed bone. It is assumed to be normal cortico-cancellous bone integrating with the implant, but without biopsies we cannot be certain of the tissue quality or the presence of any residual graft in microscopic terms. Despite these limitations, our findings suggest that placing DDM at the implant apex during a crestal sinus lift is a reliable and effective technique within its indicated range, consistent with previous research demonstrating the osteoinductive potential of demineralized dentin matrix in sinus augmentation [19, 25, 31]. The regenerated bone at the sinus floor was consistently sufficient to cover a substantial portion of the implant apex, and implant success was maintained over multiple years. Future prospective studies, ideally randomized controlled trials, are warranted to compare the use of DDM against other graft materials or even no graft in transcrestal sinus floor elevation. Such studies could more definitively elucidate the specific benefits of using DDM and help refine guidelines for when grafting is truly necessary in crestal sinus lift procedures.

## Conclusion

Within the limits of this retrospective study, the placement of demineralized dentin matrix at the implant apex during crestal sinus floor elevation proved to be a safe and effective technique. Implants placed with this approach showed a 100% survival rate over a mean 4-year period, with no significant sinus complications. Augmenting the transcrestal sinus lift with a small autogenous DDM graft at the implant apex combines the osteogenic stimulus of an autograft with the minimally invasive nature of the crestal approach. This appears to promote reliable bone formation around the implant tip even when that tip protrudes into the sinus cavity.

## Data Availability

The datasets used and/or analyzed during the current study available from the corresponding author on reasonable request.
